# Continuous Glucose Monitors and Activity Trackers to Inform Insulin Dosing in Type 1 Diabetes: The University of Virginia Contribution

**DOI:** 10.3390/s19245386

**Published:** 2019-12-06

**Authors:** Chiara Fabris, Basak Ozaslan, Marc D. Breton

**Affiliations:** Center for Diabetes Technology, University of Virginia, 560 Ray C Hunt Dr, Charlottesville, VA 22903, USA; bo3rp@virginia.edu (B.O.); mb6nt@virginia.edu (M.D.B.)

**Keywords:** continuous glucose monitors, activity trackers, smart insulin dosing, type 1 diabetes

## Abstract

*Objective*: Suboptimal insulin dosing in type 1 diabetes (T1D) is frequently associated with time-varying insulin requirements driven by various psycho-behavioral and physiological factors influencing insulin sensitivity (IS). Among these, physical activity has been widely recognized as a trigger of altered IS both during and following the exercise effort, but limited indication is available for the management of structured and (even more) unstructured activity in T1D. In this work, we present two methods to inform insulin dosing with biosignals from wearable sensors to improve glycemic control in individuals with T1D. *Research Design and Methods*: Continuous glucose monitors (CGM) and activity trackers are leveraged by the methods. The first method uses CGM records to estimate IS in real time and adjust the insulin dose according to a person’s insulin needs; the second method uses step count data to inform the bolus calculation with the residual glucose-lowering effects of recently performed (structured or unstructured) physical activity. The methods were tested in silico within the University of Virginia/Padova T1D Simulator. A standard bolus calculator and the proposed “smart” systems were deployed in the control of one meal in presence of increased/decreased IS (*Study 1*) and following a 1-hour exercise bout (*Study 2*). Postprandial glycemic control was assessed in terms of time spent in different glycemic ranges and low/high blood glucose indices (LBGI/HBGI), and compared between the dosing strategies. *Results*: In Study 1, the CGM-informed system allowed to reduce exposure to hypoglycemia in presence of increased IS (percent time < 70 mg/dL: 6.1% versus 9.9%; LBGI: 1.9 versus 3.2) and exposure to hyperglycemia in presence of decreased IS (percent time > 180 mg/dL: 14.6% versus 18.3%; HBGI: 3.0 versus 3.9), tending toward optimal control. In Study 2, the step count-informed system allowed to reduce hypoglycemia (percent time < 70 mg/dL: 3.9% versus 13.4%; LBGI: 1.7 versus 3.2) at the cost of a minor increase in exposure to hyperglycemia (percent time > 180 mg/dL: 11.9% versus 7.5%; HBGI: 2.4 versus 1.5). *Conclusions*: We presented and validated in silico two methods for the smart dosing of prandial insulin in T1D. If seen within an ensemble, the two algorithms provide alternatives to individuals with T1D for improving insulin dosing accommodating a large variety of treatment options. Future work will be devoted to test the safety and efficacy of the methods in free-living conditions.

## 1. Introduction

In health, blood glucose (BG) levels are maintained within a safe range (typically 70–180 mg/dL) by a complex neurohormonal network that acts toward minimizing hypoglycemia (i.e., BG < 70 mg/dL) and attenuating exposure to hyperglycemia (i.e., BG > 180 mg/dL). Insulin is the primary regulator of glucose homeostasis, and the glucose and insulin subsystems interact via feedback control signals to quickly respond to glycemic perturbations (e.g., following meals) and bring BG to the pre-perturbation level [1]. In type 1 diabetes (T1D), the sophisticated mechanisms regulating glucose homeostasis are degraded because of the autoimmune destruction of insulin-producing pancreatic β-cells, which renders internal insulin secretion practically absent [2]. Consequently, individuals with T1D are unable to endogenously counteract BG perturbations and need to rely on exogenous insulin to control their BG levels. 

The treatment of T1D is generally based on insulin replacement strategies driven by frequent BG monitoring. Insulin therapy commonly implements multiple daily insulin injections, combining short- and long-acting insulin analogs, or continuous subcutaneous insulin infusion, where insulin pumps are programmed to almost continuously deliver small amounts of insulin, augmented by larger doses around meals [3]. To inform insulin dosing decisions, sparse self-monitoring of capillary glucose (SMBG) or continuous (i.e., up to every five minutes) monitoring of interstitial glucose (CGM) provide alternatives to individuals with T1D for monitoring their BG levels [4,5]. 

Despite the improving accuracy of BG monitoring devices [5,6] and the growing development of decision support systems [7,8], suboptimal insulin replacement remains common in T1D, leading to excess mortality and complication rates that are still significantly higher when compared to the general population [9,10]. In fact, the quality of glycemic control in individuals with T1D is heavily dependent on multiple daily treatment decisions by the patients accounting for a variety of factors influencing insulin demand (e.g., circadian rhythms, physical activity, and psychological stress). The main mediator of time-varying insulin requirements is insulin sensitivity (IS), a metabolic parameter describing how sensitive the body is to the action of insulin, which summarizes into a single number the effect of increasing plasma insulin on the enhancement of glucose uptake and inhibition of endogenous glucose production [11,12,13]. In order to compensate for systematic intraday IS variations, physicians periodically review SMBG or CGM traces to adjust basal rate, insulin-to-carbohydrate ratio (CR), and correction factor (CF) profiles. Early research is trying to automatize this task by developing algorithms for titrating individual therapy parameters, including learning approaches with structured SMBGs [14,15,16,17,18] and CGM-based decision support systems capable of providing feedbacks to clinicians regarding suggested therapy changes [7,19,20,21]. 

While systematic IS variations can be compensated through optimally tuned time-varying treatment parameters, superimposed IS fluctuations triggered by acute, non-systematic events are more challenging to handle and further complicate insulin dosing. Among the various psycho-behavioral and physiological factors shown to influence insulin requirements [22,23,24,25,26], physical activity remains a major trigger of altered IS both during and following the exercise bout [27,28,29,30,31,32]. Due to the relevant BG changes accompanying exercise sessions, the American Diabetes Association has released guidelines suggesting that people with T1D adjust their insulin dose and carbohydrate intake to account for physiological changes resulting from structured exercise bouts [33]. However, these guidelines are neither intended to nor can provide optimal BG management for every person, since the optimal timing, direction, and magnitude of treatment adjustment can vary by person and their respective situations [31]. Moreover, the range of physical activity influencing BG metabolism in T1D is broader than structured exercise, with even light, unstructured activity having a significant impact on BG levels [34,35,36,37]. 

Whether triggered by a structured exercise session, a light walk, or a situation of increased psychological stress, episodic IS changes represent a common source of imperfect insulin replacement, typically leading to worsened quality of glycemic control and higher glucose variability (GV). However, “smart” bolus calculators informed by estimated time-varying insulin needs could allow for the tailoring of prandial insulin doses to the metabolic state of a person, therefore mitigating the impact of IS fluctuations on GV and quality of glycemic control. 

In this work, we present an ensemble of two methods developed by our group to optimize insulin dosing in individuals with T1D by informing the standard insulin bolus calculation with signals collected in real-time from wearable sensors. The first method relies on CGM data from minimally invasive glucose sensors and uses an estimate of the subject’s current IS state to modulate the insulin dose; the second method uses physical activity data from ubiquitous activity trackers to inform the bolus calculation with recently performed (structured or unstructured) physical activity. The design of two methods driven by different biosignals aims at accommodating the variety of treatment options offered to individuals with T1D, providing an alternative to people not using a CGM system. While human clinical trials are needed to test the safety and efficacy of the designed smart bolus calculators in real-life scenarios, here we present the results from an in-silico validation of the methods within the University of Virginia (UVA)/Padova T1D simulation platform [38]. Specific scenarios are built to test the algorithms within their domain of application, and the smart systems are compared to standard bolus therapy in terms of postprandial GV and quality of glycemic control.

## 2. Research Design and Methods

### 2.1. CGM Data to Inform Insulin Dosing

In presence of highly informative CGM measurements and insulin records, real-time IS assessments can be obtained and leveraged to inform insulin dosing [7]. Specifically, we developed a method to estimate IS that relies on CGM data and a linear time-invariant model of glucose-insulin dynamics embedded within an optimally tuned Kalman filter (KF). The KF implementation allows to track IS in real time by estimating a new IS value every five minutes, as a new CGM data point becomes available. Insulin pump records (basal/bolus and meal information) are needed to reconstruct the KF inputs through models of subcutaneous insulin transport and meal absorption. The smart insulin bolus is then computed by modulating a standard bolus with the ratio of usual IS estimated from historical data, and a real-time IS assessment computed on demand at the time of the bolus administration. In a real-life scenario, the IS tracking algorithm can be run on CGM and insulin records gathered over several weeks (e.g., four) of home monitoring; a 24-hour median IS profile can then be built for each person, which is representative of his/her usual intraday pattern of sensitivity to insulin action. The profile embeds IS fluctuations driven by circadian rhythms and systematic behavioral habits, and is assumed to be compensated for by individual insulin treatment parameters routinely optimized by treating physicians. After computing the IS profile, indicating with B the standard bolus, the IS-informed insulin dose (B_IS_) is calculated in real time as

(1) $$B=\frac{\mathrm{CHO}}{\mathrm{CR}}+\frac{\mathrm{BG}-{\mathrm{BG}}_{\mathrm{TGT}}}{\mathrm{CF}}-{\mathrm{IOB}}_{\mathrm{BOL}}\;B_{\mathrm{IS}}=\frac{{\mathrm{IS}}_{\mathrm{PRF}}}{{\mathrm{IS}}_{\mathrm{RT}}}\left(\frac{\mathrm{CHO}}{\mathrm{CR}}-\frac{\mathrm{BG}-{\mathrm{BG}}_{\mathrm{TGT}}}{\mathrm{CF}}\right)+{\mathrm{IOB}}_{\mathrm{BAS}}\left(\frac{{\mathrm{IS}}_{\mathrm{PRF}}}{{\mathrm{IS}}_{\mathrm{RT}}}-1\right)-{\mathrm{IOB}}_{\mathrm{BOL}}$$

where CHO is the amount of meal carbohydrates, BG is the prevailing CGM value at mealtime, BG_TGT_ is the glycemic target, IOB_BOL/BAS_ is the insulin on board (IOB) from bolus (BOL) and basal (BAS) injections, and IS_RT/PRF_ are the IS estimates computed in real time (RT) and drawn for the profile at a comparable time of day (PRF). Based on the design of the IS-informed bolus calculator, the system administers a larger insulin dose if an IS level lower than usual is detected, while the bolus is reduced in presence of heightened IS; of note, if the real-time IS estimate equals the estimate from the profile, then B_IS_ naturally converges to B.

### 2.2. Physical Activity Data to Inform Insulin Dosing 

This method uses physical activity data collected at frequent intervals to inform the bolus decision with changes in insulin needs due to recently performed physical activity. In this work, step count traces are used to track the amount of physical activity performed over time; however, the method could be easily adjusted to work with any other activity-related signal (e.g., heart rate). Given step count data, an indicator of accumulated physical activity can be calculated at any time of day as a weighted sum of previously walked steps; this indicator is referred to as activity on board (AOB) and translates the prolonged glycemic impact of physical activity into a single number quantifying the residual glucose-lowering effects of previously performed activity. Leveraging the AOB concept, the physical activity-informed insulin bolus is computed at the time of a standard meal (e.g., breakfast, lunch, dinner) by adjusting the standard bolus based on the deviation between real-time AOB and routine AOB for the corresponding meal calculated from historical data. In a real-life scenario, the routine AOB profile is a representation of the physical activity that a person usually accumulates by the time of each standard meal and can be obtained by evaluating several weeks of step count data collected through a pedometer or an off-the-shelf activity tracker. The AOB profile embeds the glycemic impact of systematically performed, routine activity, which is assumed to be accounted for by individual insulin treatment parameters. Therefore, the activity-informed insulin dosing method adjusts the insulin bolus only when the accumulated physical activity deviates from the daily routine. After extracting the AOB profile value for the standard meal associated with a certain bolus B, the physical activity-informed insulin dose (B_PA_) is calculated as:

(2) $$B_{\mathrm{PA}}=B-\frac{{\mathrm{AOB}}_{\mathrm{RT}}-{\mathrm{AOB}}_{\mathrm{PRF}}}{\mathrm{AF}}$$

where AOB_RT_ is the real-time AOB assessment, AOB_PRF_ is the routine AOB from the profile previously computed for the corresponding standard meal, and AF is the subject-specific activity factor, which converts the AOB deviation from profile into insulin unit equivalents and can be optimized for each person based on historical data. As mentioned for the CGM-driven method, based on the design of the physical activity-informed bolus calculator, the system administers a larger insulin dose if a person has been less active than usual in the hours preceding a certain meal, while the bolus is reduced if the person has been more active than usual.

### 2.3. Multi-Source Smart Insulin Dosing

**Figure 1:**
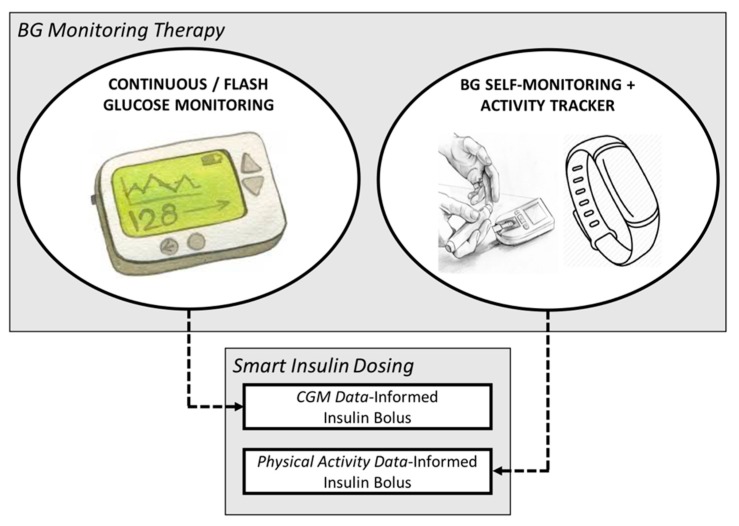
Diagram representing the ensemble of the two designed methods that renders smart insulin dosing flexible to individual therapy policies.

The ensemble of the two presented methods is designed to accommodate the variety of treatment options available to individuals with T1D. As shown in Figure 1, in presence of highly informative CGM data, a precise estimate of IS can be obtained in real time and used to inform the insulin dose. On the other hand, for patients not using a CGM device and relying on SMBG therapy, a simple activity tracker can be sufficient to mitigate the effects of the main trigger of altered IS, i.e., physical activity. 

### 2.4. Simulation Studies

Two simulation studies were run within the UVA/Padova T1D Simulator [38], respectively designed to test each of the proposed smart bolus calculators. The two studies are described below.

Study 1: CGM-Informed Bolus Calculator

A single-meal scenario was simulated for 100 virtual adults under three IS conditions: (a) nominal IS; (b) increased IS; and (c) decreased IS. The IS variation was simulated for each subject randomly sampling from a normal distribution centered around the subject’s nominal IS value with a 40% coefficient of variation. For scenarios (b) and (c), the same simulation was repeated twice, and the meal was bolused according to the standard or smart bolus calculator, defined above as B and B_IS_, respectively. Postprandial glycemic control was assessed for the increased/decreased IS scenarios and the two meal bolusing strategies in terms of percent time in different glycemic ranges (i.e., < 70 mg/dL and > 180 mg/dL) and low/high BG indices (LBGI/HBGI – GV indicators introduced in [39,40,41] and described below). The glycemic outcomes were then compared between the two runs of simulations (i.e., standard versus smart bolus calculator) and against the optimal control achieved in the nominal case.

Study 2: Step Count-Informed Bolus Calculator

A 31-hour study was run in the same population of 100 virtual adult subjects. Across the study, subjects received three meals (breakfast-lunch-dinner) at 07:00, 13:00, and 19:00, respectively containing 40, 70, and 60 grams of carbohydrates. At 16:00, a 1-hour moderate-intensity exercise bout was simulated, which was designed to alter the subject’s IS level for up to 12 hours following the bout [27]. For each virtual adult, a step count profile was generated according to the average number of steps per minute reported in resting and running conditions [42]. No steps were assumed to happen overnight, between 21:00 and 06:30; in the resting state (i.e., from 06:30 to 21:00, except from exercise), an average of 8 steps/minute was assumed; during exercise, an average of 140 steps/minute was simulated. Inter-subject variability during rest and exercise was generated according to uniform distributions centered around the reported average step counts, with 35% and 10% variability, respectively. Throughout the entire simulation, hypoglycemia treatments were administered at BG < 60 mg/dL during rest and BG < 80 mg/dL during exercise; after a hypoglycemic event, BG was checked every 20 minutes and treatment was repeated if hypoglycemia was not resolved. For each subject, the simulation was repeated twice, deploying the standard or step count-informed bolus calculator for the control of the dinner meal. Postprandial glycemic control was then assessed in terms of percent time spent in different glycemic ranges, as well as LBGI and HBGI, and compared between the two sets of simulations.

### 2.5. Glycemic Risk Indices: LBGI and HBGI

LBGI and HBGI are previously introduced GV measures designed to aggregate the frequency and extent of low and high BG events, respectively, into single numbers [39,40,41]. The computation of LBGI and HBGI requires a symmetrization of the BG range based on a logarithmic transformation, which is then used to associate a measure of hypoglycemic or hyperglycemic risk to each collection of BG measurements. In formulas, the BG risk function is defined as follows

(3) $$r\left(\mathrm{BG}\right)=10\cdot 1.509^{2}\cdot {\left\{{\left[\log\left(\mathrm{BG}\right)\right]}^{1.084}-5.381\right\}}^{2}$$

with low-BG and high-BG risk functions further defined as

(4) $$r_{\mathrm{LOW}}\left(\mathrm{BG}\right)=\{\begin{array}{ll} r\left(\mathrm{BG}\right), & \mathrm{if}\;\mathrm{BG}<112.5\;\mathrm{mg}/\mathrm{dL}\\ 0, & \mathrm{if}\;\mathrm{BG}\geq 112.5\;\mathrm{mg}/\mathrm{dL} \end{array}$$

(5) $$r_{\mathrm{HIGH}}\left(\mathrm{BG}\right)=\{\begin{array}{ll} 0, & \mathrm{if}\;\mathrm{BG}<112.5\;\mathrm{mg}/\mathrm{dL}\\ r\left(\mathrm{BG}\right), & \mathrm{if}\;\mathrm{BG}\geq 112.5\;\mathrm{mg}/\mathrm{dL} \end{array}$$

which allow to define LBGI and HBGI as

(6) $$\mathrm{LBGI}=\frac{1}{N}\sum_{i=1}^{N}r_{\mathrm{LOW}}\left({\mathrm{BG}}_{i}\right)$$

(7) $$\mathrm{HBGI}=\frac{1}{N}\sum_{i=1}^{N}r_{\mathrm{HIGH}}\left({\mathrm{BG}}_{i}\right)$$

where the average extends to all the BG samples within the considered collection of measurements. By this definition, a higher LBGI may indicate a large number of mild hypoglycemic events, a small number of significant events, or a combination of both; and the same applies to HBGI with respect to hyperglycemia.

## 3. Results

Throughout this section, results obtained with the use of the standard bolus calculator are reported in light gray, while results obtained with the use of either proposed smart system are reported in dark gray.

Study 1: CGM-Informed Bolus Calculator

**Figure 2:**
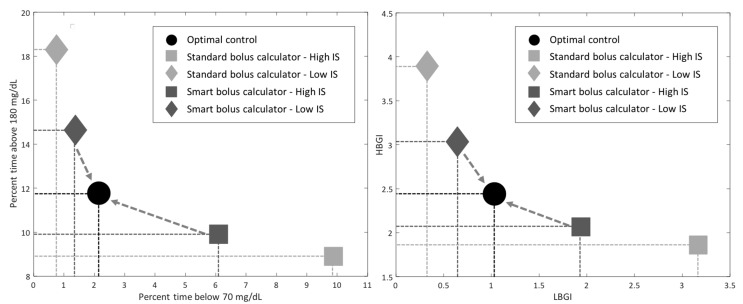
*Study 1 results*: Postprandial exposure to hypoglycemia (x-axis) and hyperglycemia (y-axis) obtained with the use of the standard (light gray) and smart (dark gray) bolus calculator in presence of increased (square) and decreased (diamond) insulin sensitivity (IS), as compared to the optimal control achieved in nominal IS conditions (black circle).

**Figure 3:**
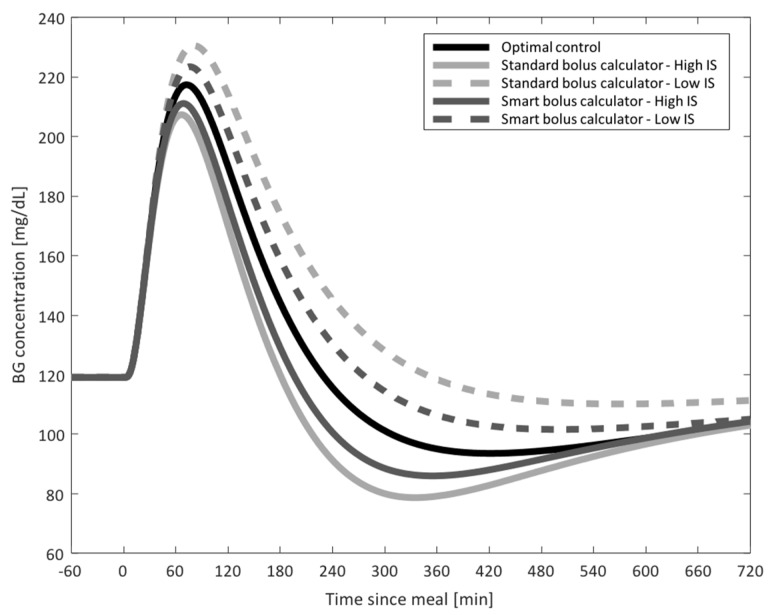
*Study 1 results*: Average blood glucose (BG) profiles obtained with the use of the standard (light gray) and smart (dark gray) bolus calculator for the increased (solid) and decreased (dotted) insulin sensitivity (IS) scenarios, as compared to optimal control (solid black).

IS was estimated in the hours preceding the controlled meal to determine the value of IS_RT_; IS_PRF_ was considered to be the IS value estimated for each subject in the nominal IS scenario. Performances of the standard and smart bolus calculator in presence of decreased/increased IS are shown in Figure 2, as compared to the glycemic control achieved with optimal therapy parameters in the nominal IS case. In presence of higher sensitivity, the IS-informed bolus calculator allowed to achieve a relevant reduction in exposure to hypoglycemia (percent time < 70 mg/dL: 6.1% versus 9.9%; LBGI: 1.9 versus 3.2), without increasing hyperglycemia (percent time > 180 mg/dL: 9.9% versus 8.9%; HBGI: 2.1 versus 1.9). Likewise, when the sensitivity was lower than usual, the smart system decreased exposure to hyperglycemia (percent time > 180 mg/dL: 14.6% versus 18.3%; HBGI: 3.0 versus 3.9), without relevant increase to hypoglycemia (percent time < 70 mg/dL: 1.4% versus 0.8%; LBGI: 0.7 versus 0.3). This is observable also in Figure 3, where the average BG profiles are shown for the different scenarios and prandial insulin dosing policies. From both figures, it is visible how the use of the smart system allows to handle IS fluctuations, improving the quality of postprandial glycemic control and tending toward the performance of optimal insulin therapy parameters.

Study 2: Step Count-Informed Bolus Calculator

**Figure 4:**
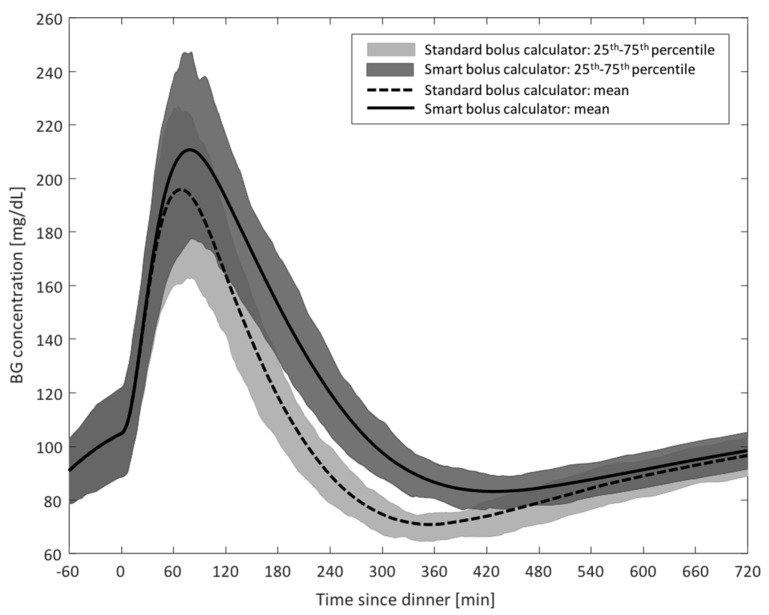
*Study 2 results*: Envelope of postprandial blood glucose (BG) traces obtained with the use of the standard (dotted line and light gray) and smart (solid line and dark gray) bolus calculator following a 1-hour aerobic exercise session.

**Figure 5:**
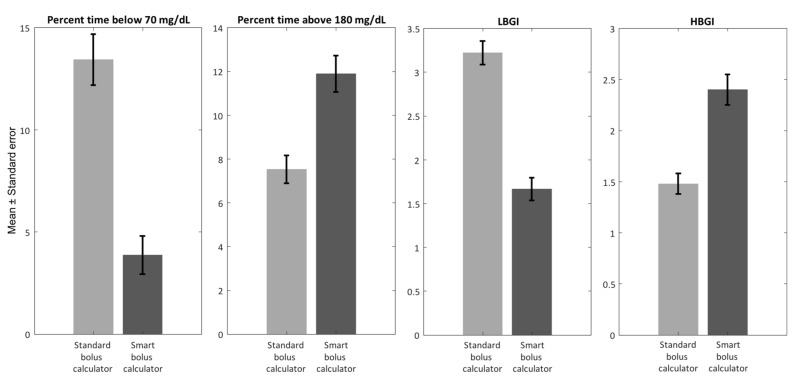
*Study 2 results*: Summary of postprandial GV indices obtained with the use of the standard (light gray) and smart (dark gray) bolus calculator following a 1-hour aerobic exercise session.

Each subject’s AOB_RT_ was computed at the time of the dinner meal and compared to the AOB_PRF_ value previously calculated assuming that the subject did not perform the 1-hour exercise session. A population value for AF was used, set to 3000 steps/U. Figure 4 shows the envelope of the postprandial BG traces obtained following a dinner bolus administered based on the standard or smart bolus calculator; Figure 5 presents a summary of postprandial GV metrics. As visible from the figures, the use of the smart bolus calculator reduced postprandial hypoglycemia (percent time < 70 mg/dL: 3.9% versus 13.4%; LBGI: 1.7 versus 3.2) at the cost of a minor increase in exposure to hyperglycemia (percent time > 180 mg/dL: 11.9% versus 7.5%; HBGI: 2.4 versus 1.5). In addition, the use of the smart system allowed to reduce the number of hypoglycemia treatments administered following dinner from 35 to seven.

## 4. Conclusions

Suboptimal insulin dosing in T1D is at the root of individuals’ inability to reduce GV and achieve good glycemic control, therefore representing a source of long-term complications. The periodic review of glycemic profiles computed manually by treating physicians or automatically through learning methods and ad hoc algorithms [7,14,15,16,17,18,19,20,21], allows for the optimization of insulin treatment parameters to account for systematic intraday IS patterns. However, insulin requirements in T1D are highly variable and impacted by several physiological and psycho-behavioral factors affecting IS [22,23,24,25,26]. Among these, physical activity remains a major challenge for individuals with T1D [28,29,30,31,32], with aerobic exercise having a delayed impact on individuals’ sensitivity to insulin action lasting for several hours past the conclusion of the activity bout [27].

In this work, we presented two methods developed at the University of Virginia to inform insulin dosing in individuals with T1D based on biosignals easily collectable through wearable sensors. The first method relies on highly informative data from CGM systems; using CGM records and a model of glucose–insulin interaction embedded within an optimally tuned KF, the method allows to track IS on demand and inform the bolus calculation with the real-time assessment of a person’s insulin requirements. The second method does not use glycemic profiles and relies on physical activity data collected from ubiquitous activity trackers to inform the bolus calculation. Specifically, the method tracks the amount of physical activity performed by a person in the hours before a meal and adjusts the prandial insulin dose taking into account the remaining glucose-lowering effects of previously performed structured and unstructured physical activity (here measured in terms of step count). The two methods serve different needs, and the aim of this paper is not to compare their performance. On the contrary, if considered within an ensemble, the two methods provide smart insulin dosing alternatives to individuals with T1D accommodating a wide range of treatment strategies: in presence of CGM data, the first method offers an accurate estimates of a person’s insulin needs and can inform the bolus calculation to compensate for IS variations triggered by many psycho-behavioral and physiological factors; on the other hand, for patients not using a CGM device and relying on SMBG therapy, the use of a simple activity tracker can be leveraged to inform insulin dosing and mitigate the effects of structured and unstructured physical activity on GV and quality of glycemic control. The methods, as tested in silico within the UVA/Padova T1D Simulator, showed promising performance in terms of improved postprandial glycemic control compared to standard bolus calculation.

The major limitation of the current study is inevitably related to the limited intra- and inter-subject variability provided by the UVA/Padova Simulator. Therefore, the mandatory next step entails testing the proposed technology in real-life scenarios within human clinical trials. In addition, both methods proposed here rely on the patient’s therapy parameters. In simulation, optimal CR, CF, and basal rates are available for each virtual subject; however, in a real-life application, assuming optimal therapy parameters may not be plausible. Therefore, further analyses will be needed to assess the robustness of the proposed algorithms to suboptimal CR/CF configurations and the possibility of combining the techniques with ad hoc optimizers of insulin therapy parameters.
